# How Work Intensification Relates to Organization-Level Safety Performance: The Mediating Roles of Safety Climate, Safety Motivation, and Safety Knowledge

**DOI:** 10.3389/fpsyg.2018.02575

**Published:** 2018-12-17

**Authors:** Johanna Bunner, Roman Prem, Christian Korunka

**Affiliations:** ^1^Department of Applied Psychology: Work, Education, Economy, Faculty of Psychology, University of Vienna, Vienna, Austria; ^2^Work and Organizational Psychology, Institute of Psychology, University of Graz, Graz, Austria

**Keywords:** safety professionals, safety engineers, managers, serial mediation, high-accident

## Abstract

Recent changes in the world of work have led to increased job demands with subsequent effects on occupational safety. Although work intensification has been linked to detrimental safety behavior and more accidents, there is so far no sufficient explanation for this relationship. This paper investigates the mediating roles of safety climate, safety motivation, and safety knowledge in the relationships of work intensification with components of safety performance at an organizational level. Safety engineers and managers from 122 Austrian high-accident companies participated in a cross-sectional survey. In line with our hypotheses, work intensification negatively related to both components of safety performance: safety compliance and safety participation. The results of a serial multiple mediation analysis further revealed safety climate and safety motivation to be serial mediators of the relationship between work intensification and safety performance. Unexpectedly, safety knowledge and safety climate only serially mediated the relationship between work intensification and safety compliance, but not the relationship between work intensification and safety participation. This study provides evidence for the detrimental effect of work intensification on safety performance across organizations. Additionally, this study offers an explanation as to how work intensification affects safety performance, enabling practitioners to protect their occupational safety procedures and policies from work intensification.

## Introduction

The intensification of work has been one of the most significant changes in the world of work since the 1980s (European Agency for Safety and Health at Work [EASHW], 2007). The acceleration of economic and technological development through new technologies (Rosa, 2003) both requires and enables companies to be more efficient in the production of goods and in the delivery of services. Due to increased economic pressure, fewer employees must do more work more frequently in less time. Although automation and digitalization facilitate faster decision processes and shorter production cycles, halts in production owing to maintenance, human error, or unforeseen events threaten output goals and can generate high expenses for companies. This increases the pressure on employees to work at a higher speed (Green, 2004) and/or reduce their breaks (Roberts, 2007). Ultimately, this culminates in work intensification which, in addition to work and time pressure, requires employees to continuously invest more work effort to complete more work in less time (Green and McIntosh, 2001).

Empirical evidence suggests that work intensification has adverse effects on occupational safety and health. On an individual level, several studies have found that common job demands such as work overload and time pressure encourage unsafe work practices (Slappendel et al., 1993; Hofmann and Stetzer, 1996; Mullen, 2004; Hansez and Chmiel, 2010). Work intensification, however, is different from such job demands and refers to the process of continuously increasing job demands that have to be attended to in shorter time (Green and McIntosh, 2001; Green, 2004; Kubicek et al., 2015). Although it has been argued that there is insufficient statistical data to prove the causal negative or positive effects of work intensification on health and well-being in the workplace (European Agency for Safety and Health at Work [EASHW], 2007), recent findings suggest that work intensification adversely affects individual occupational safety (Koukoulaki, 2010; Papadopoulos et al., 2010; Akamangwa, 2016; Walters and Wadworth, 2017).

As work intensification stems from societal developments leading to organizational adjustments (Green, 2004), its impact has been found across a variety of organizations such as hospitals, hotels, nursing homes, offices, and construction sites as well as in manufacturing productions (Mullen, 2004; European Agency for Safety and Health at Work [EASHW], 2007; Oxenbridge and Moensted, 2011; Kubicek et al., 2013, 2015; Paškvan et al., 2016). To our knowledge, there are no studies yet that have explored how work intensification affects safety performance across several organizations. Therefore, the present study investigates the effects of work intensification on safety performance at the organizational level. To explain the relationship of work intensification and safety performance across organizations, we draw on the model of safety performance (Griffin and Neal, 2000; Neal and Griffin, 2002, 2004), which has been tested at the organizational level in a study based on expert evaluations (Braunger et al., 2015). It describes the effect of safety climate on safety performance by considering the mediating roles of safety motivation and safety knowledge. We assume that the negative relationship of work intensification with safety performance, consisting of safety compliance and safety participation, can be explained via its adverse impact on safety climate. Based on the model of safety performance, we will test serial indirect effects between work intensification and safety performance via safety climate and consequently, both, safety motivation and safety knowledge at the organizational level.

The present study contributes to the literature on safety performance in two ways. First, we demonstrate that the effect of work intensification on safety performance can be found across a variety of organizations. This finding indicates that the detrimental effects of work intensification may be a challenge of societal origin rather than due to organizations’ failure. Second, drawing on the model of safety performance allows us to reveal how the relationship of work intensification with the components of safety performance can be explained via safety climate and the determinants of safety performance. This furthers the understanding of the mechanisms of work intensification at an organizational level.

### Work Intensification and Safety Performance

Work intensification refers to the increasing amount of effort an employee must invest during the working day that oftentimes results from increased economic pressure and other societal changes (Green and McIntosh, 2001; Green, 2004). In contrast to time pressure, resulting from high quantitative workload at a specific point in time, work intensification refers to increasing levels of quantitative workload over time. In other words, work intensification is characterized by an increased need to complete more tasks within one working day, work at a heightened speed, perform different tasks simultaneously, and/or reduce idle time (Kubicek et al., 2014, 2015).

When production output is at risk, employees are often expected to work faster rather than safe (Paté-Cornell, 1990; Zohar, 2008). Thus, work intensification contributes to the safety-production conflict, which is a persistent topic in occupational safety research. This manifests in employees trading quality for quantity of work (Amabile et al., 1976), to meet production goals. Especially in the production sector, work intensification is associated with a decline in occupational safety and a rise in musculoskeletal disorders and other psychosocial risks (European Agency for Safety and Health at Work [EASHW], 2007). Additionally, work intensification leads to the use of unsafe working methods and consequently, also more injuries (Oxenbridge and Moensted, 2011) because employees who experience work intensification are more likely to by-pass and block safety systems of machineries to work faster and maintain production rates (European Agency for Safety and Health at Work [EASHW], 2007; Koukoulaki, 2010). These negative safety behaviors, driven by work intensification, are indicators of poor safety performance.

Safety performance can be broadly defined as actions or behaviors that individuals carry out in their jobs to ensure their individual health and safety as well as that of their surrounding environment (Burke et al., 2002). It plays a crucial role in maintaining a safe work environment as it has been shown to predict workplace injuries (Neal and Griffin, 2006; Christian et al., 2009) and accordingly, higher levels of safety performance are associated with fewer occupational injuries (Clarke, 2013). The distinction between safety compliance and safety participation as components of safety performance was first conceptualized by Neal and Griffin (2002). Safety compliance refers to generally mandated core activities carried out by individuals to maintain their personal workplace safety such as wearing personal protective equipment or complying with safety procedures at work. In contrast, safety participation describes individuals’ behavior that contributes to developing a safe work environment. These behaviors include, for example, participation in voluntary safety activities, the attendance of safety meetings, and helping coworkers with safety-related issues. Safety participation, in contrast to safety compliance, does not immediately contribute to the individuals’ safety but rather to the overall safety of the workplace (Neal and Griffin, 2002, 2006; Griffin and Curcuruto, 2016).

Individuals engage in safety compliant behavior because it is required of them but also to protect themselves from accidents and injuries. Work intensification, however, might undermine this behavior. Meta-analytic evidence suggests that work intensification as a result of down-sizing can be expected to have negative effects on occupational injury and safety compliance (Quinlan and Bohle, 2009). High levels of work intensification result in workarounds, such as bypassing or neutralizing the safety systems of machinery and the negligence of other protective equipment. This occurs because disregarding safety rules may be the only way for employees to sustain heightened work speed and maintain production rates (Askenazy, 2001; Burchell, 2006; European Agency for Safety and Health at Work [EASHW], 2007; Cavazza and Serpe, 2009). Thus, when work intensification is high, complying with safety regulations may require greater effort than non-compliance (Tregaskis et al., 2013). Overall, findings suggest that work intensification is associated with lower levels of safety compliance.

Work intensification might also compromise employees’ participation in safety behavior contributing to a safe work environment. As work intensification increases, a decline in safe work behavior as well as training and information regarding safety can be observed (Koukoulaki, 2010; Papadopoulos et al., 2010). With tightly scheduled production, the time for workers to attend safety meetings are cut short, as they must fulfill production goals. Additionally, when workers learn that adverse safety behavior is the most efficient way to reach production goals, they will most likely refrain from telling each other off for not working safely and thus not participate in creating a safe work environment. Additionally, work intensification has been linked to poor communication at work, leading to a subsequent loss of values and respect for colleagues (European Agency for Safety and Health at Work [EASHW], 2007) and to occupational violence, harassment, and bullying (European Foundation for the Improvement of Living and Working Conditions. [EFILWC], 2002; Quinlan and Bohle, 2009). Working in an environment of bullying and harassment not only impairs mental health with detrimental consequences such as anxiety, dysphoria, and reduced self-confidence or even a decrease in organizational commitment (Nielsen and Einarsen, 2012; Giorgi et al., 2016). Thus, poor communication may also reduce instances of workers helping each other to work safely or investing in the company’s safety voluntarily. Overall, it can be expected that work intensification is negatively related to both safety compliance and safety participation. Thus, we propose the following relationships:

**H1.** Work intensification is negatively related to the components of safety performance, i.e., (a) safety compliance and (b) safety participation.

### The Roles of Safety Climate, Safety Motivation, and Safety Knowledge

Safety climate describes perceptions of safety in the work environment regarding the priority of safety during production processes involving physical or health risks (Zohar, 2000, 2014) that are shared within an organization (Griffin and Neal, 2000). The role of organizational safety climate has been investigated extensively and literature shows that it significantly affects employees’ motivation to work safely, their execution of compliant and participative safety behavior, and safety outcomes such as accidents and injuries (e.g., Christian et al., 2009; Nahrgang et al., 2011). Especially in the context of the model of safety performance, safety climate plays a significant role. The priority of safety policies, procedures, and practices in the form of safety climate informs employees to what extent their workplace supports and rewards safety compliant or engaging behavior and thus, drives their motivation to behave safely and sustain their safety knowledge (Neal et al., 2000; Neal and Griffin, 2006; Zohar, 2014; Braunger et al., 2015).

On a conceptual level, safety climate is a second-order factor that is derived from the following first-order factors: safety practices, management values, safety equipment, safety training, and safety communication (Griffin and Neal, 2000; Neal and Griffin, 2004; Braunger et al., 2015). In the subsequent section, we argue that work intensification negatively affects the first-order factors of safety climate and consequently, negatively relates to safety climate.

Detrimental effects of work intensification on safety practices often remain unnoticed because work intensification stems from structural and organizational changes of work such as more flexible organization structures and the reduction of the workforce (Cascio, 2003). Thus, organizations overlook that safety practices and OSH (occupational safety and health) workplace standards are negatively affected by work intensification (Quinlan and Bohle, 2009; Walters and Wadworth, 2017). Work intensification also has adverse effects on safety climate in that it is negatively correlated with employees’ perception of the organizations’ safety concerns (Cavazza and Serpe, 2009). Management values and managerial commitment toward safety are a crucial aspect of safety climate that influence it strongly (Zohar, 2008). Additionally, work intensification has been found to cause poor communication and a low social climate at work (European Agency for Safety and Health at Work [EASHW], 2007) which may affect safety communication. Because safety communication has no direct and visible contribution to the organizations output, it may seem futile to exhaust organizational resources on it, when those are direly needed to reach production goals. Likewise, safety training requires financial, temporal, and human resources. Work intensification, however, necessitates that fewer employees accomplish more work in less time, thus, cutting resources for other activities scarce. Unsurprisingly, work intensification is often accompanied by a reduction in permanent staff and an increase in contract workers who receive fewer instructions and less training (Koukoulaki, 2010; Papadopoulos et al., 2010). Lastly, when work demands and production speed increase and there is no leeway in production schedules, employees refuse to wear their personal protective equipment if it hinders them to work more efficiently (Turner et al., 2005; Ahmed et al., 2015) or slows down their work speed (Forst et al., 2006). The extent to which an organization tolerates improperly adjusted safety devices and invests into employees′ personal safety equipment contributes to the level of safety climate in that organization (Zohar, 2014). As work intensification appears to be negatively associated with each of the five aspects of safety climate, we assume that it is also negatively related to safety climate.

According to the model of safety performance (Griffin and Neal, 2000; Neal et al., 2000; Neal and Griffin, 2002, 2004, 2006) safety climate affects both, safety motivation and safety knowledge. These determinants of safety performance, in turn, are related to both, safety compliance, and safety participation. The relationship between the antecedents, determinants, and components of safety performance has been well-researched and confirmed (e.g., Clarke, 2000; Griffin and Neal, 2000; Neal et al., 2000; Neal and Griffin, 2002, 2004, 2006; Christian et al., 2009; Braunger et al., 2015; Griffin and Curcuruto, 2016).

**FIGURE 1:**
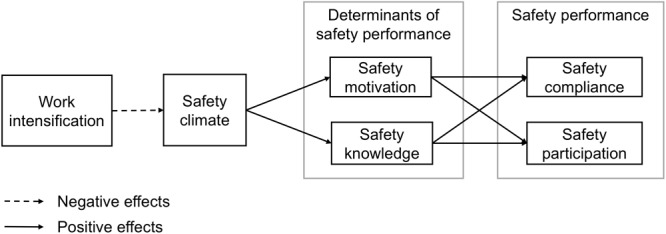
Conceptual model of the relationships between work intensification on safety compliance and safety participation via safety climate and safety motivation resp. safety climate and safety knowledge.

Safety motivation can be described as an individual’s willingness to participate in safety activities and to comply with safe working practices. It has been conceptualized as a key determinant of safety compliance and safety participation across a range of industrial and organizational contexts (Griffin and Curcuruto, 2016). Griffin and Neal (2000) assumed that safety climate as a distal factor of safety performance has an indirect effect on safety compliance and safety participation by influencing employees’ safety motivation. The effect of safety climate on safety performance through safety motivation can be explained by both expectancy-valence-theory (Vroom, 1964) and social exchange theory (Blau, 1964). According to expectancy-valence-theory, the level of safety climate determines which safety behaviors are reinforced. Thus, a positive safety climate reinforces expectancy-value perceptions of safety behaviors (Parker et al., 2010) and motivates employees to execute the valued behaviors (Zohar, 2010). Additionally, in line with social exchange theory (Blau, 1964), when employees perceive that the organization supports and cares about their well-being, they will reciprocate by enacting behaviors that benefit the organization. Thus, safety motivation should lead to safety behaviors that keep the individual worker safe but also contribute to a safe work environment. Therefore, we assume the following relationship (see Figure 1):

**H2.** The negative relationship of work intensification with the components of safety performance, i.e., (a) safety compliance and (b) safety participation, is serially mediated by safety climate followed by safety motivation.

Safety knowledge refers to an individual’s knowledge and skills of how to comply with safety regulations or participate in safety activities in order to stay safe (Neal and Griffin, 2002). Safety climate may influence safety performance through safety knowledge because relevant knowledge and skills are required to carry out safe work behaviors. An employee without sufficient knowledge of how to comply with safety regulations or participate in safety activities will not be able to perform these actions (Neal and Griffin, 2002). Through the level of safety climate they perceive, employees are informed about the emphasis the organization puts on said knowledge and skills of how to work safely (Zohar, 2014). Thus, we assume the following relationship (see Figure 1):

**H3.** The negative relationship of work intensification with the components of safety performance, i.e., (a) safety compliance and (b) safety participation, is serially mediated by safety climate followed by safety knowledge.

### Investigating the Relationship of Work Intensification and Safety Performance at the Organizational Level

Work intensification impacts the individuals’ health and safety. However, it stems from societal changes and its detrimental effects have been found in numerous organizations. Thus, we assume it also has an impact at the organizational level that is manifested in organizational safety climate. As safety climate is a specific form of organizational climate (Neal et al., 2000) it may be impacted by work intensification which stems from societal developments and leads to organizational changes (Green and McIntosh, 2001; Green, 2004). This assumption is supported by findings from Lindell and Brandt (2000) that showed how organizational climate is affected by environmental context variables.

Testing this assumption with data from multiple organizations allows for a higher variance. While employee data is often aggregated to explore the organizational level, this approach seems unfit when aiming for data collection across many organizations. For this intention, a currently very encouraging approach in safety research is to utilize safety expert and manager perspectives (Nordlöf et al., 2012, 2017; Braunger et al., 2015). Experts, such as safety engineers, have a better representation of tasks within their area of expertise and they encode new information more efficiently that allows them access to information and knowledge relevant to the demands of action in current situations and tasks. Accordingly, safety engineers’ ratings of organizational-level safety correlate highly with employees’ aggregated perceptions of safety climate (Zohar, 1980). In the field of occupational health and safety, however, both managers and safety professionals are apt to evaluate changes in work demands and aspects of occupational safety. Previous research has shown that even though their perceptions may differ on how pronounced certain work and safety situations are, they still positively correlate as managers and safety experts agree on the most important work and safety issues and thus, their prioritization (Nordlöf et al., 2012, 2017).

## Materials and Methods

### Study Design

Data was collected during consulting sessions that the Austrian Workers’ Compensation Board [Allgemeine Unfallversicherungsanstalt (AUVA)] provided free of charge to high-accident companies. In a cross-sectional design, safety engineers, and managers were asked to anonymously answer an online-questionnaire during the consultation. Complementary data regarding company size and sector according to NACE classification (EUROSTAT, 2008) was provided by the AUVA. This study was carried out in accordance with the recommendations of the American Psychological Association [APA] (2010). The procedure and materials of this study have not undergone examination by an ethics committee, as the measures and procedures followed the protocols of standard survey study research in applied psychology and we did not touch sensitive topics (like, e.g., sexual orientation). Our protocol fully complied with the standards of the university where it was conducted. These standards include strict guidelines to store potentially identifying information like e-mail addresses separately from the focal measures. Individuals interested in participating in our study were informed about the general aims and the protocol of the study before their participation. At the beginning of the online questionnaire participants were informed about the confidentiality of the study and their voluntary participation. They had the option to quit the survey at any time. To start survey participation, they had to tick a box on the landing page of the online questionnaire, stating they had understood and agreed with the conditions of the survey.

### Description of Respondents

Safety engineers and managers were chosen because their expert evaluation provides an organizational perspective of working and safety conditions in each organization. Respondents were asked to state their age and tenure in the organization. Safety engineers also stated whether they provided their services externally or were employed as in-house experts. Managers were asked about their position in the company. The sample consisted of 51.6% safety engineers, most of them were employed as in-house experts. The managers had an average tenure of 10.7 years in different management positions, supervising 77 employees on average. Table 1 provides more detailed descriptions of the respondents.

**Table 1:** Description of respondents’ role, age, employment status, or position.

		*n*	%
Respondents’ role		122	
	Safety engineers	63	51.6
	Managers	59	48.4
Age	21–30 years	6	5.3
	31–40 years	20	17.7
	41–50 years	55	48.7
	Above 51 years	32	28.3
	Missing	9	
Safety engineers’ status	In-house	35	55.6
	External	28	44.4
	Missing	1	
Managers’ position	CEO	15	25.4
	Site manager	8	13.5
	Production manager	10	17.0
	HR manager	10	17.0
	Division manager (other)	14	23.7
	Group managers	2	3.4

		***M***	

Organizational tenure	Safety engineers	11.3 years	
	Managers	10.7 years	

### Sample and Data Collection

Although the occurrence of workplace accidents in the European Union and Austria has been decreasing during the last 20 years, there are still high-accident companies. They appear to be especially challenged with balancing the competing goals of productivity and safety (Paté-Cornell, 1990; Zohar, 2000) and thus, comprise an attractive sample when investigating the impact of detrimental working conditions on occupational safety. In Austria, a company is considered high-accident when it can be listed among the 1000 companies with the highest accident-per-employee-rate in any given year. These high-accident companies are offered optional free consultations by the AUVA. From January 2016 to December 2016 we were able to conduct a survey in 122 of these companies receiving a free consultation. Either a safety engineer or a manager representing their company during the consultation completed the complementary questionnaire of this study. During data collection, the AUVA-headquarter sent out monthly reminders to their regional managers with the request to encourage consultants to promote study participation during the consultations. At the beginning of the consultation, participants were invited to voluntarily answer the questionnaire and ensured that their data would be processed anonymously.

Access to the online questionnaire required log-in with the companies’ insurance number which was later also used to match the descriptive company data. Most of the organizations had between 51 and 250 employees and the biggest sectors were the manufacturing of goods and construction. A detailed overview of the companies’ sectors and number of employees is provided in Table 2. Table 2 also contains the distribution of the sectors and sizes of all high-accident companies of 2015. The sample’s distribution of sectors is representative for manufacturing of goods and construction. The distribution of our samples’ company sizes are not representative of the high-accident companies of 2015. However, the accident-per-employee rate of the sample is representative for Austrian high-accident-companies of the same year. The sample’s mean accident-per-employee rate was 0.67 reportable accidents-per-employee in 2015 with a range from 0.06 to 2.18. The accident-per-employee rate for all Austrian high-accident companies in 2015 was 0.60 and ranged from 0.16 to 2.36 accidents-per-employee.

**Table 2:** Description of companies’ sizes and sectors.

		Sample (*N* = 122)		High-accident companies 2015
	
		*n*	%	%
Size	1–50 employee(s)	0	0.0	0.0
	51–250 employees	89	72.9	47.2
	251 and more employees	33	27.1	52.8
Sector	Manufacturing of goods	54	44.3	40.3
	Construction	26	21.3	28.2
	Trade and maintenance	7	5.7	11.3
	Traffic and warehousing	5	4.1	7.2
	Agriculture, forestry, fishery	4	3.3	0.7
	Water supply, sewage and waste disposal, removal of pollution	4	3.3	1.9
	Other sectors	22	18.0	10.4

### Measures

In advance, the questionnaire was discussed with a focus group of managers and safety engineers to ensure equal comprehensibility for all respondents. All measures were administered in German.

#### Work Intensification

Work intensification was measured using the intensification of job demands scale by Kubicek et al. (2015). In this scale, an impersonal formulation of items is used to measure perceived changes in job demands. Because we measured at an organizational level, the original items had to be slightly adapted to facilitate judgment of work intensification at the organizational level. One example is the adaptation from “In the past 2 years…it is increasingly harder to take time for breaks” to “In the past 2 years…it is increasingly harder for the employees of this organization to take time for breaks.” The response format was a five-point Likert-scale ranging from 1 (*not at all*) to 5 (*completely*).

#### Safety Climate, Safety Motivation, Safety Knowledge, Safety Compliance, and Safety Participation

Safety climate, safety motivation, safety knowledge, safety compliance, and safety participation were assessed using the safety climate questionnaire by Braunger et al. (2015). This questionnaire was designed specifically for safety engineers to give their expert evaluation on how they perceive organizational safety climate as well as the determinants and components of safety performance, allowing for the collection of data across many organizations. The discussion in the focus group served to ensure managers’ comprehensibility and evaluative competencies of these items. All items were answered on a five-point Likert-scale with the alternatives ranging from 1 (*strongly disagree*) to 5 (*strongly agree*).

*Safety climate* was assessed using a 10 item-questionnaire with two items each measuring the first-order factors management values, safety practices, safety communication, safety training, and safety equipment. Safety engineers and managers were asked how they evaluated the organization regarding those aspects. An example item for safety communication is “The goals, measures, and facilities concerning workplace safety are public or made available to all employees in the organization.”

*Safety motivation* was assessed with two items referring to the employees’ willingness to comply with safety regulations. The items were “The employees in this organization try to minimize the danger of accident in their workplaces” and “Workplace safety has high importance by the employees of this organization.”

*Safety knowledge* was assessed with two items asking about employees’ knowledge of safety procedures and practices. The items were “The employees in this organization know about the hazards in their workplaces” and “The employees in this organization know how they can work safely.”

*Safety compliance* was assessed with two items relating to employees’ main activities for maintaining their individual safety. The items were “The employees in this organization always work according to safety rules, even under time pressures” and “The employees in this organization always wear the protective equipment or clothing.”

*Safety participation* was assessed with two items that referred to the employees’ behavior that contributes to a safety-supporting environment. The items were “The employees in this organization take part in the development and implementation of initiatives within workplace safety” and “The employees in this organization voluntarily attend safety trainings.”

#### Control Variables

We controlled for number of employees per organization and the respondents’ role (safety engineer or manager). There is empirical evidence that safety engineers and managers agree on general improvements in the field of work environment management, an effect that can also be found over time (Swedish Work Environment Authority, 2006, 2008; Nordlöf et al., 2012). Some literature, however, indicates that safety engineers and managers perceive the company’s priority for work and safety environment management moderately different (Forth et al., 2006; Nordlöf et al., 2012). As there is no conclusive empirical evidence that managers and safety engineers agree in their description of safety and working conditions or employee behavior, we decided to include the professional role of the respondents as a control variable in the model.

### Data Analysis

Figure 1 illustrates the proposed relationships between the variables. All hypotheses were tested using standardized variables. We tested our hypotheses simultaneously using structural equation modeling (SEM) in Mplus 7.4 (Muthén and Muthén, 1998–2015). Safety climate was included as a latent variable measured by five indicators: management values, safety training, safety communication, safety practices, and safety equipment. All other variables in the model were included as manifest variables. With SEM, multiple complex relationships between a set of variables can be analyzed simultaneously. Moreover, a variable can be both, a dependent variable in one relationship and a predictor variable in another relationship at the same time (i.e., a mediator). In serial mediation, a specified direction of causal flow is assumed in which the mediators are linked. The presumed direction of the flow is usually based on theoretical justification and decided by the researcher (Hayes, 2017). Mplus tests all possible variable combinations for the specific ordering and, as a result, provides all direct and indirect effects for the tested model. Because the distribution of serial indirect effects is skewed in most cases, we obtained bootstrapped 95% bias-corrected confidence intervals (CIs) based on 10,000 draws. As recommended by Becker (2005), we tested our model with and without control variables.

## Results

### Descriptive Statistics

The internal consistencies were satisfactory for work intensification, safety climate, safety motivation, and safety knowledge (α ≥ 0.83). Reliabilities for safety compliance and safety participation were found to be acceptable (α ≥ 0.72). Most variables were correlated with each other, except for the company size, which only correlated with the respondents’ role, and work intensification with safety motivation. The means, standard deviations, correlations, and internal consistencies (Cronbach’s α) for all variables are provided in Table 3.

**Table 3:** Descriptive statistics, correlation coefficients, and internal consistencies for all variables (*N* = 122).

	Measure	*M*	*SD*	1	2	3	4	5	6	7	8
1	No. of employees	183.60	203.86								
2	Respondents’ role	1.48	0.50	**–0.19**							
3	Work intensification	1.89	0.75	0.09	**–0.18**	(0.89)					
4	Safety climate	4.26	0.57	0.12	**0.26**	**–0.28**	(0.89)				
5	Safety motivation	4.03	0.77	0.03	**0.36**	–0.12	**0.56**	(0.87)			
6	Safety knowledge	4.27	0.65	–0.15	**0.31**	**–0.38**	**0.53**	**0.57**	(0.83)		
7	Safety compliance	3.83	0.77	–0.08	**0.39**	**–0.39**	**0.69**	**0.70**	**0.66**	(0.72)	
8	Safety participation	3.68	0.89	–0.06	**0.32**	**–0.27**	**0.63**	**0.56**	**0.42**	**0.62**	(0.72)

*Correlation coefficients significant with *p* < 0.01, two-tailed, are shown in boldface. Cronbach’s α are shown in the diagonal in parentheses. Respondent’s role: 1 = safety engineer, 2 = manager.*

### Hypotheses Testing

We tested all hypotheses simultaneously in a SEM controlling for the number of employees and the respondents’ role within the respective organization. Analyses were repeated without control variables, but interpretation of the hypothesis stayed identical. The model showed a good fit with χ^2^ = 63.2, df = 33, RMSEA = 0.08 [0.05; 0.12], CFI = 0.95, SRMR = 0.04, TLI = 0.91.

**FIGURE 2:**
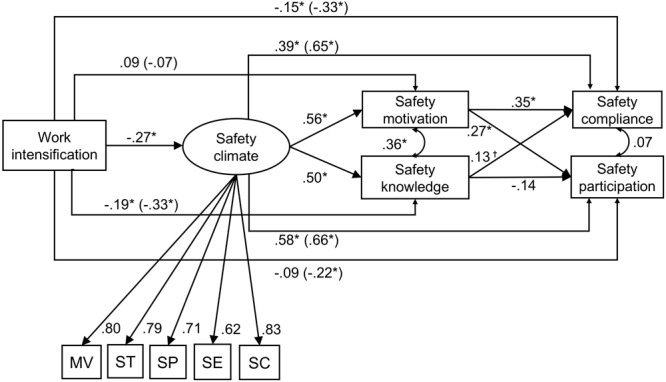
Standardized estimates and total effects from structural equation modeling (SEM). ^†^*p* < 0.10; ^∗^*p* < 0.05. Total effects are displayed in parentheses. For visual clarity control variables are omitted. MVs, management values; ST, safety training; SPs, safety practices; SE, safety equipment; SC, safety communication.

The control variables had relationships with some of the variables. The number of employees was positively related to safety climate β = 0.21, 95% CI [0.04; -0.33] and negatively to safety knowledge β = -0.18, 95% CI [-0.29; -0.05]. The respondent’s role was negatively related to safety climate β = -0.27, 95% CI [-0.44; -0.09] and safety motivation β = -0.22, 95% CI [-0.37; -0.06]. Work intensification was directly negatively related to safety climate, safety knowledge, and safety compliance. Safety climate was directly positively related to safety motivation, safety knowledge, safety compliance, and safety participation. Safety motivation was positively related to both, safety compliance and participation, whereas safety knowledge was not significantly related to safety participation and only showed a positive significant relationship to safety compliance with *p* < 0.10. Figure 2 illustrates the estimates for the direct and total effects from the SEM.

Hypotheses 1ab proposed negative relationships between work intensification and safety compliance as well as between work intensification and safety participation. Consistent with Hypotheses 1a and 1b, work intensification had a significant negative relationship with safety compliance β = -0.33, 95% CI [-0.47; -0.15] as well as safety participation β = -0.22, 95% CI [-0.37; -0.05]. Thus, Hypotheses 1a and 1b were supported.

Hypotheses 2ab predicted indirect serial effects of work intensification via safety climate and safety motivation on the two outcomes, safety compliance and safety participation. As the CI of the indirect effect of work intensification via safety climate and safety motivation on safety compliance did not include zero, Hypothesis 2a was supported. There was also a significant indirect effect of work intensification via safety climate and safety motivation on safety participation. Thus, Hypothesis 2b was supported as well. Hypotheses 3ab predicted serial indirect effects of work intensification via safety climate and safety knowledge on safety compliance and safety participation. Even though safety knowledge was only significantly related to safety compliance within a 90% bias-corrected CI β = 0.134, 90% CI [0.01; 0.257], the indirect effect was significant, Hypothesis 3a was supported. Hypothesis 3b, however, had to be rejected, because safety knowledge did not show a significant relationship with safety participation and consequently, the estimate of the respective serial indirect effect was not significantly different from zero. Table 4 provides an overview of the standardized indirect and total effects of the analyses for all hypotheses.

**Table 4:** Standardized indirect and total effects of main effects and mediation analyses.

	Estimate	Bias-corrected bootstrap 95% confidence interval	
		LL	UL
**Total effects**			
H1a: WI → SCo	**–0.33**	–0.477	–0.158
H1b: WI → SP	**–0.22**	–0.372	–0.047
**Indirect effects**			
H2a: WI → SC → SM → SCo	**–0.05**	–0.199	–0.012
H2b: WI → SC → SM → SP	**–0.02**	–0.059	–0.003
H3a: WI → SC → SK → SCo	**–0.04**	–0.104	–0.010
H3b: WI → SC → SK → SP	0.02	–0.002	0.070

*Significant effects are shown in boldface. WI, work intensification; SC, safety climate; SM, safety motivation; SK, safety knowledge; SCo, safety compliance; SP, safety participation.*

## Discussion

In this study, we proposed a relationship between work intensification and safety performance at the organizational level that we explained based on the model of safety performance (Griffin and Neal, 2000; Neal et al., 2000). We tested our hypotheses based on data provided by either safety engineers or managers from 122 high-risk organizations.

### Work Intensification and Safety Performance

In line with our hypotheses building on previous studies (European Agency for Safety and Health at Work [EASHW], 2007; Quinlan and Bohle, 2009; Tregaskis et al., 2013), we found that work intensification was negatively related to safety compliance and safety participation at the organizational level. To our knowledge, this is the first study to provide empirical evidence for the relationship between work intensification and safety participation at the organizational level. These findings suggest that work intensification not only compromises individual safety behavior but also the safety of work environments within an organization. Using the perspective of one representative per organization to assess levels of work intensification and safety performance allowed us to show the negative impact of work intensification across high-risk organizations of different sizes and sectors. This may lead to the conclusion that work intensification as a societal issue has detrimental effects on occupational safety that expand beyond the scope of individual safety behavior or the safety measures of single organizations.

### The Role of Safety Climate, Safety Motivation, and Safety Knowledge

We used SEM to test our assumption that the negative relationship of work intensification with safety performance can be explained through the model of safety performance (Neal et al., 2000; Neal and Griffin, 2002, 2004). By drawing from this model, we incorporated safety climate as a first stage mediator and safety motivation and safety knowledge as second stage mediators in the relationship between work intensification and safety performance. Examining these relationships from an organizational level allowed us to theoretically account for the fact that work intensification stems from structural and organizational changes of work (Cascio, 2003). These assumptions were largely supported in our study.

As expected, work intensification was strongly related to safety climate. The results suggest that work intensification not only directly negatively affects safety performance but also indirectly affects it through worsening the organizations’ safety climate. Safety climate, in turn, directly and indirectly affects safety performance. These findings are consistent with the notion that safety climate is an indicator of production over safety; a low safety climate meaning that there is a higher focus on production (Zohar, 2014). On a broader level, it is important to recognize that safety climate is a specific form of organizational climate (Zohar, 1980). With work intensification resulting from organizational and structural changes (Cascio, 2003), other forms of organizational climate may also be affected by work intensification. Future research could examine adverse effects of work intensification on different types of climate, such as absence or ethical climate.

As shown in previous studies (Griffin and Neal, 2000; Neal et al., 2000; Braunger et al., 2015), safety climate affects safety compliance and participation through a positive impact on safety motivation. In our study, we could show that this association persists at an organizational level when work intensification was added as a predictor. In this relationship, the role of safety climate is essential. Although work intensification had a direct negative effect on safety climate, it had no direct effect on safety motivation. This indicates that workload and time pressure in the form of work intensification do not lead to employees seeming less motivated in their efforts to minimize the danger of their work or think of safety as unimportant. Instead, changes in organizational safety climate strongly affect safety motivation. Accordingly, safety motivation showed a direct positive relationship with both safety compliance and safety participation. Thus, understanding what motivates employees to work safely is of utmost importance when striving to reinforce safety compliant and participative behaviors.

Safety knowledge and safety climate acted as serial mediators in the relationship of work intensification and safety compliance but not in the relationship of work intensification and safety participation. In one of their earlier studies, Griffin and Neal (2000) found that safety knowledge was more strongly related to safety compliance than safety participation. They argued that it was more important for an individual to know how to work safely to comply with safety rules than it was for their involvement in participatory activities. In line with these assumptions, safety knowledge was not significantly related with safety participation in our analysis. However, it should be noted that both variables shared a positive and significant zero-order correlation. Their relationship only became non-significant in the more complex model of our analyses when other variables predicting safety participation were included.

We also unexpectedly found that work intensification had a strong negative association with safety knowledge that was not explained through safety climate. This association can be explained by previous research which has shown that an increase in work intensity is often accompanied by a decrease in communication, training and information regarding safety (Koukoulaki, 2010; Papadopoulos et al., 2010) which reduces the safety knowledge among employees. Additionally, antecedents and by-product of work intensification, such as downsizing and restructuring or tightened production schedules and increased workload, may also contribute to a loss of institutional safety knowledge. When managers or experienced workers leave the company without being replaced, it is possible that crucial safety information is being lost. Also, when employees must take on more and new tasks to compensate for a reduced workforce, they might not be provided with sufficient safety information to carry out all of their tasks safely (Sauter et al., 2002).

### Strengths and Limitations

This study utilized the evaluation of one safety engineer or manager per organization rather than measuring employee perceptions on work intensification and occupational safety. This can be considered both, a strength as well as a limitation. In contrast to the conventional method of aggregating individual data to the group and organizational levels, having organizational representatives or experts evaluate organizational conditions and characteristics can provide some advantages. First, picking the appropriate experts for the study can produce more unbiased information. We chose safety engineers and managers for their relevant insight into organizational work and safety practices. With safety engineers, for example, it can be assumed that they base the organizational evaluation on their specific expertise and as such, provide an impartial assessment (Wu et al., 2007; Fernández-Muñiz et al., 2009; Braunger et al., 2015). Second, it is more efficient and thus, economical if just one representative per organization is required for the evaluation. For instance, employing multiple participants to gather data about an organization might be possible in large companies, however, this approach is more difficult when surveying smaller ones (Rindfleisch et al., 2008). Thus, the approach allows researchers to investigate the development and pattern of a widespread phenomenon over numerous companies.

Even though literature has found that the perspectives of safety professionals and managers positively correlate and they generally agree on the most important work and safety topics, some research argues that safety engineers and managers perceive their organization’s priority for work and safety through different lenses (Forth et al., 2006; Nordlöf et al., 2012, 2017). Following the recommendations of Becker (2005) on how to handle statistical control variables in organizational research, we included the respondents’ role as a control variable to account for the different perspectives and because it was correlated with the dependent variables safety compliance and safety performance. Furthermore, we tested the hypothesized model with and without control variables and although the respondents’ role was positively related to safety climate and safety motivation, model results were essentially the same. Therefore, in this study, the respondents’ role can be ruled out as a confounding variable.

Of course, using expert evaluations is a limited approach, as it, for example, is not appropriate for assessing an individual’s perceptions, beliefs, judgments, or feelings (Podsakoff et al., 2012). We measured how safety engineers and managers perceived employees’ safety motivation and safety knowledge. Naturally, neither safety engineers nor managers can read employees′ minds to assess their degree of safety knowledge or motivation. Therefore, safety motivation items were phrased in a descriptive way asking to report observable behavior rather than requesting participants to speculate about employees′ intentions or degree of suspected motivation. Both, safety engineers and managers are mainly responsible for employees’ safety instructions and trainings, thus for the transmission of safety knowledge (Braunger et al., 2009, 2015). Therefore, they are eligible to judge the employees’ state of safety knowledge. Additionally, we controlled for the respondents’ position to account for possible different perspectives.

The measures in our study showed as good as, if not better, internal consistencies compared to the original publications (Braunger et al., 2015; Kubicek et al., 2015). This can be attributed to the conceptualization of our constructs. All items were phrased descriptively and thus, in a way a third person with insight into the organization could provide their evaluation. Work intensification as well as safety climate is related to developments in organizational structures and practices that are observable from an outside perspective. Because work intensification relates to objective rather than subjective changes in job demands (Kubicek et al., 2015), it is apt to be assessed by experts at an organizational level. Investigating numerous organizations in one study enabled us to develop a broad perspective on the occurrence of work intensification across high-accident organizations. This also holds true in the context of occupational safety. While safety climate refers to shared perceptions of the priority of safety within one organization, this value of safety is deducted from observable safety measures and actions. The actions set to create safety climate are of utmost importance as they strongly indicate the relevance of safety within the organization (Zohar, 2014). As such, our safety climate measure relates to organizational safety policies and procedures that affect safety behavior; both are observable from an outside perspective.

Our study can only reveal associations between work intensification, safety climate, safety motivation, safety knowledge, and safety performance due to its cross-sectional design. To show the causal relationships between these constructs, a longitudinal design is necessary. Longitudinal studies could determine whether work intensification reduces safety performance over time and determine the long-term roles of safety climate, safety knowledge, and safety motivation play in this model. It would be advisable for future research to incorporate four measurement points in their study designs to test causal effects in the serial mediations and to avoid common method bias (Podsakoff et al., 2003, 2012).

While our sample is representative for Austrian high-accident organizations regarding their sectors and accident-employee-ratio, it is not representative regarding the number of employees. In our analyses, however, we controlled for the number of employees. Yet, it remains unclear whether these results can be replicated in less accident-prone organizations. Future researchers should investigate whether our outcomes are valid in countries with similar legal embedding of organizations and design of OSH structures.

### Practical Implications

The results have important practical implications for organizations. When work intensification increases, organizations may have to expect two kinds of adverse consequences regarding safety. First, on an individual level, employees’ safety performance may decrease because of intensified working conditions, enhancing the risk of accidents and injuries. Especially behaviors that contribute to a safe work environment may decrease owing to work and time pressure, worsening employees’ cohesion, and overall safety. Secondly, managers must be made aware of these risks and be actively responsible for creating an environment where safe work behavior is incentivized, as their safety values are a driver for positive safety climate.

Additionally, our results indicate that work intensification also negatively affects safety climate and safety knowledge. In an environment where production is more important than safety, time, and other resources for significant safety activities may be cut short. Organizations need to be aware that work intensification has detrimental effects on occupational safety practices and procedures. They may prevent this deterioration by (a) taking countermeasures to uphold a positive level of safety climate and (b) providing their employees with the necessary resources to enable them to safely conduct their work. From our results, it can be argued that it may be especially effective to invest in employees’ safety knowledge as it is directly affected by work intensification. Promising measures to counteract the detrimental effects of work intensification include safety instructions, training, and clear communication of practices and procedures. Especially in highly intensified work environments, these trainings should be administered in frequent, short intervals to enhance employees’ familiarity with new safety strategies. This is important because work teams are prone to return to less effective strategies that they are more familiar with when time pressure is high (Lehner et al., 1997). Naturally, even short regular trainings of employees are time and cost intensive. Current research suggests that training supervisors’ safety awareness is a very effective and resource efficient way of incentivizing employees to work safely. Supervisors and managers act as multipliers in their teams and through their role behavior motivate employees to work safely (Luria, 2016). Especially in organizations with high work intensification, sending supervisors to train rather than a whole team might be a more cost-sensitive option.

Employees may experience work intensification because organizations are laying off their colleagues or replace them with contract workers to cut costs. Organizations also employ such strategies when trying to survive during economic crises. Amid recession, resources are shifted away from OSH measures because organizations are focused on restructuring and downsizing (International Labour Organization [ILO], 2013; Jhang, 2018). While the detrimental effects of crises (or the fear thereof) on mental and physical health of employees are well-researched (Giorgi et al., 2015; Mucci et al., 2016), it appears that empirical research findings on the causal links between recession and higher levels of occupational accidents are inconclusive (International Labour Organization [ILO], 2013; de la Fuente et al., 2014). Nevertheless, crises have been found to increase the workload for OSH professionals, especially under the light of recruitment freezes for OSH staff. Additionally, priorities are shifted away from OSH, resulting in negative management attitudes toward OSH which negatively impacts safety culture (International Labour Organization [ILO], 2013; Jhang, 2018). Therefore, it is thinkable that economic crises also compromise safety climate, safety motivation and safety knowledge of workers at an organizational level because it leads to similar reactions across organizations. Managers signaling low importance of safety during crises impair their safety climate and thus, decrease their employees’ motivation to work safely. Furthermore, minimizing OSH measures reduces safety training which results in lower safety knowledge among employees. In turn, employees with little safety knowledge and motivation will behave less safely. While the effects of economic crises on safety climate and safety behavior have not been thoroughly researched, there is reason to suspect that they are similar to those of work intensification.

It is essential that worker safety is always a priority for organizations and they invest sufficient resources in preventative measures. To protect their employees from work intensification and its detrimental consequences, organizations may stop laying off employees to cut costs temporarily, replacing them with contract workers. Furthermore, keeping experienced employees in the company will protect safety standards and facilitate the training of newer employees. Organizations may also keep their employees safe and healthy by protecting their break times and keeping work teams at a size that allows for a manageable workload.

## Conclusion

This study showed that work intensification negatively relates to safety compliance and safety participation at the organizational level. Additionally, it shows how these relationships are mediated by safety climate and safety motivation and that safety climate and safety knowledge also mediate the relationship between work intensification and safety compliance. Thus, this study makes two contributions to the literature. First, it is the first study to show a direct negative relationship between work intensification and safety performance. In addition, it provides evidence for the detrimental effect of work intensification on safety performance at an organizational level, showing that work intensification is a challenge of societal origin rather than organizations’ failure. Second, this is the first study to offer an explanation as to how work intensification affects safety behavior. By drawing from the model of safety performance, we further the understanding of the mechanisms behind the extensive effects work intensification has on occupational safety. Additionally, this study expands research on expert perspectives by entirely utilizing the perspective of safety engineers and managers to evaluate work intensification and organizational safety variables.

## Author Contributions

JB and CK organized the collection and preparation of data. JB and RP performed the statistical analyses, wrote the first draft of the manuscript, and made changes to the manuscript during the interactive review stage. All authors contributed to the conception and design of the study, read and edited the manuscript, and suggested improvements at several stages during the preparation and revision of the manuscript.

## Conflict of Interest Statement

The authors declare that the research was conducted in the absence of any commercial or financial relationships that could be construed as a potential conflict of interest.
